# Making the best better: performance psychology integrated in special operation forces training

**DOI:** 10.3389/fpsyg.2025.1537792

**Published:** 2025-03-26

**Authors:** Christian Ytterbøl, Dave Collins, Alan MacPherson

**Affiliations:** ^1^Military Academy, Norwegian Defence University College, Oslo, Norway; ^2^Moray House School of Education and Sport, University of Edinburgh, Edinburgh, United Kingdom

**Keywords:** elite military, mental skills training, performance under pressure, special operation forces, performance psychology

## Abstract

**Introduction:**

This study investigates how performance psychology was employed in conjunction with an operational combat squadron in NORSOF (Norwegian Special Operation Forces) whilst they were preparing for deployment. Access to this group allowed the research team to evaluate the contribution which a performance psychology package (PP) can make to an elite military unit with little or no previous exposure of such training.

**Methods:**

The study utilized an explorative longitudinal case study format to evaluate a mixed intervention. Content was delivered in two formats: psychoeducation in a classroom setting and *in situ* PP delivery during training. Data were collected through four stages of semi-structured, in-depth interviews with a significant time interval between each stage. Stage one occurred prior to the intervention—offering an informal baseline to understand what knowledge and skills the operators perceived in themselves; Stage two was conducted prior to deployment, after the unit's workup had finished. Stage three data was gathered upon the unit's return from deployment. Finally, as stage 4, an 18-month follow-up was conducted with key members of the unit.

**Results:**

Analyzed through Reflective Thematic Analysis, results indicate that the operators already held a high level of mental skills—likely a result of absorbing practices that are learned and developed in order to adapt successfully to their performance environment. Importantly, however, results also indicate that the additional structured PP intervention, integrated within the unit's workup training, increased perceived performance at both an individual and unit level. The follow-up supports the initial results. Discussion: Although SOF already are high performers and very good at what they do, our contention is that performance psychology could be a valuable and important concept to integrate and develop further.

## Introduction

The development of psychological performance constructs through mental skills training (MST) is increasingly popular (Birrer and Morgan, 2010; Chesterton et al., 2020). Importantly, these methods are seen not only as adjuncts, but also as essential and causative precursors to enhanced performance in sports and related performance domains (Anton et al., 2016; Pecen et al., 2016; Spoon et al., 2020).

Reflecting this, a significant amount of work has been undertaken in challenging environments; for example, Special Weapons and Tactics or SWAT teams (Andersen et al., 2015; Liu et al., 2018). Within the general military context, informative research is emerging on providing training that can improve the increasingly popular construct of resilience (Cornum et al., 2011; Jones et al., 2023; Precious and Lindsay, 2019; Williams et al., 2016). In addition, a wide range of different MST interventions have been conducted within the general military context (Fitzwater et al., 2018; DeWiggins et al., 2010; Jensen et al., 2020; Meyer, 2018). Work conducted to-date on SOF has largely focused on preliminary evaluations of possible MST interventions (Mattie et al., 2020), specific elements such as personnel selection (Pattyn et al., 2024), or physical training (Solberg et al., 2015). Whilst informative, work to date reflects a gap in the research. In light of this, our research on this topic has focused on three main areas. Firstly, longitudinal research conducted in partnership with SOF operators that assesses a bespoke package of performance psychology training—delivered and evaluated in conjunction with the unit and its individual operators. Secondly, understanding more about what and how—an in-depth understanding, from the perspective of the SOF operator regarding their cognition in preparation for and performance in combat. Lastly, the need for deliberate consideration of this under researched, special group of high performers (Ytterbøl et al., 2022).

Therefore, the careful bespoke design of the PP training package is an important consideration; indeed, we would question whether mainstream performance psychology can be uncritically applied to performance domains, without sufficient consideration for, in this case, the special population operating in this sphere. Therefore, the ability to pilot a PP program could provide a novel input to the field for SOF: In short, where psychology *for* performance is the primary focus (Collins and Kamin, 2012).

To enhance this focus, we employed a four-stage data collection. Interview 1 enabled us to evaluate the participants' perceptions of their existing mental skills and how these had been developed in the absence of formal training. Interview 2 provided a participant perspective on the intervention, whilst interview 3 (post-mission) offered immediate views on how useful the PP package had proven to be. Finally, interview 4 offered an 18-month follow-up: this provided a retrospective perspective—and a considered view of the applicability and utility of the intervention (all participants had remained on active duty).

Against the comparative recency of psychological interventions in this field, many high-achieving SOF performers have not experienced any training or development in this regard. Therefore, although already able, highly trained and committed individuals—in our research SOF operators were encountering PP *after* they had arrived at a high level of performance. As such, the present study also offers a valuable means of determining how various elements of PP are critically adopted in already established high-performance groups—in both the short and longer term, offering important insights for reverse application to elite sports settings (cf. Pattyn et al., 2022). In this regard, it is important to acknowledge the “already elite” nature of SOF which generates a strong psychosocial pressure toward the ongoing pursuit of excellence. We return to this later in the paper as an important contributor to the uptake of the ideas presented. Finally, such evaluations of PP interventions satisfy the recent calls in the literature to provide research into special populations (Ytterbøl et al., 2022, 2023). Therefore, and reflecting these overlapping purposes, the objectives of this explorative longitudinal case study were to:

1. Offer an evaluation of integrating a bespoke performance psychology package on SOF personnel against the challenges of immediate mission preparation and longer-term impact.
2. Evaluate the perceived effect of mental skills training on already well-established elite performers, both immediate and post-deployment—and in the longer term.

## Method

**Figure 1:**
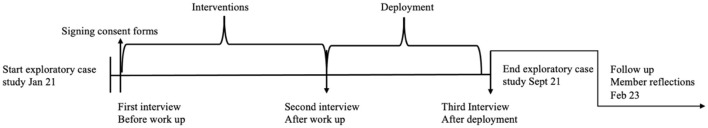
Timeline of the exploratory longitudinal case study. This figure shows significant milestones and how the investigation was operationalized. The first section contains the provision of the performance psychology package. The second part is the deployment. In addition, a follow-up was conducted 18 months after the case study ended. The total time of the study was 26 months.

Adopting a pragmatic approach (Giacobbi et al., 2005) to understand SOF operatives' work requirements, the investigation was situated in their day-to-day environment. Pragmatism indicates a concern for practical matters and is often attributed to “real world research” (Robson and McCartan, 2016, p. 28). There is already both high volume and high quality knowledge relating to the pursuit of excellence within the target group, making a methodological approach that secures their input of greater potential worth. Furthermore, pragmatism places a high regard on the reality of the inner world of human experience in action. As such, knowledge is both constructed and created in ourselves, in our social settings, and how we interact with the environment (Johnson and Onwuegbuzie, 2004). An exploratory longitudinal case study methodology (Willig, 2013; Yin, 2018) was deemed the best strategy to achieve our research goals. Regarding SOF as a novel group, this approach was adopted to understand, in-depth, how the operator's experienced PP over an extended period, both timewise and with genuine import to their performance. A mixed intervention was employed, with a blend of psychoeducation and training of the operators in their operational context. The total time from the first interaction to the final follow-up was 26 months, as per the timeline in Figure 1. Furthermore, the placement of the intervention as a structured part of their preparation for an active service deployment meant that participants would evaluate the content rigorously, offering honest and considered feedback (whether positive or negative). A Reflexive Thematic Analysis (RTA) was conducted on the content of the interviews to analyze the data (Braun and Clarke, 2019, 2023).

### Bespoke performance psychology

As the introduction explains, there is a lack of research from SOF. Therefore, the initial interview served a dual purpose. Firstly, to collect baseline data and secondly, to immediately go into specifics regarding the case conceptualization which included the task, the operator, the unit and the environment (Martindale, 2023). Initially, the interviews started at a macro level, then began to focus on tailoring potential performance interventions to this context (Collins et al., 2022). To become an operator in any SOF unit, there is a rigorous selection and training process. In addition, their culture and mission-set, place these operators in the very top echelon of military combatants' performance. Naturally, they continually evolve and most likely always want to become—even better. In our previous research, we have discussed various ways to understand this evolving process in the context of military operations (Ytterbøl et al., 2022). We would argue that the selection and subsequent training can be described as a development and learning process. It is not a training exercise you merely “endure” and then you progress into the unit. Rather, the completion of selection is the beginning of an intense period of training and deployment—and to retain a place—the commitment to continual development is a requirement; we were curious about how each operators' experiences had influenced their thoughts and behaviors across these dimensions. To understand and analyze what the operator needs in a PP program, it is vital to learn more about their thinking and how they interpreted different challenges in selection and training; and specifically, how this training influenced their own thoughts and behaviors. Given it is central to the efficacy of the training stimulus—this knowledge is held in high regard and underpins the creed of the unit. Consequently, the research team discussed and further developed the MST content based on a comparable group, namely snipers (Ytterbøl et al., 2023) whom they had worked with previously. Consequently, utilizing lived experiences, and having conducted prior research on an elite cohort of soldiers, following contact with this cohort of operators, observations were formed to develop a needs analysis which was informed by pertinent empirical and theoretical research. On the team aspect, we chose specific theoretical constructs that fits neatly in the context of practical team interaction development. In terms of the psychoeducational component of the PP, our main focus was to assist the operators to navigate the demands this taxing environment places upon them as individuals and as a team.

To teach these skills, we delivered the psychoeducation component outside the training arena, the practical application of skill occurred in a familiar, though naturally constrained environment. As a natural side effect, this enabled a Constraint Led Approach (CLA) (Hamilton et al., 2020), in the practical delivery of the PP. For example, training Close Quarter Battle could involve the team running a known or an unknown course, with lights or with night vision etc. This created a series of highly dynamic environments where the operators were subject to constraints in various ways, including scenario-based training simulating real-world challenges. In all cases, the experience was followed by a within team debrief which enabled the operators to tease out their personally generated solutions. This enabled the operators to utilize the test, tweak, repeat concept in their own development of the PP (Collins and MacNamara, 2022).

Piloting interventions in a real-world context is challenging. However, this is also where these performers train. Therefore, an important point was to actively engage in all activities performed by the troop, conducting the research with them and for them (Collins and Kamin, 2012), with the first author embedded as a performance coach.

### Participants

All participants were serving operators in the Norwegian Special Operation Forces (NORSOF). They had already successfully passed selection and completed the required training to serve as operators—albeit at different ranks and with differing specialties. All were serving operationally in a specialist troop as a part of a squadron at the time of the study (i.e., troop/company structure, but in SOF, numbers are lower than in conventional forces where a company size is usually around *n* = 150 and a troop *n* = 30).

Ethical approval from the University and local permission to conduct research in the Norwegian Armed Forces was granted. The whole troop volunteered (*n* = classified). Accordingly, to keep numbers and data collection manageable, purposive maximum variation sampling (Bryman, 2016) was conducted by the troop commander to spread age and experience within the sample evenly. Although initially selected, one participant received other orders after the first interview. As a result, the sample was reduced to seven male operators, age (*M* = 26.7, SD = 3.59) and coded *A, B, C, D, E, F, G*. Reflecting their interest and our desire to offer useful inputs, however, the whole troop received the intervention.

Prior to commencing the study, participant information letters were sent out. At the introductory session—participant information sheets were distributed. This was followed by an overview of the research project, its educational content, and the first author's role. It was made clear that the first author had two functions: a role as a performance coach and as a researcher. All participants were given a week to decide whether to take part and, if they opted in, were asked to provide written informed consent. Participation was voluntary, and all participants were assured that confidentiality and anonymity during the research process would be maintained. All participants committed to being involved, and, in addition, upon completion, the command approved the release of this research.

### Procedure

At the start of the workup period, the first author, who acted as the presenter, held three formal lectures, the longest lasting up to 40 min. The first session introduced the presenter and the research; in addition, a booklet and a PowerPoint lecture provided an overview of the performance psychology concepts to be developed (see Table 1). These were subsequently detailed in separate lectures. After this initial week, the first author followed the troop's training and became better acquainted with the participants. The first interview was conducted the following week.

**Table 1:** Overview of performance psychology components.

**Psychoeducation**	**Team development**	**Individual mental skills training**
Stress and performance in a military context (lecture and hand-out)	Professional judgement and decision-making (Collins and Collins, 2017)	Goal setting: adopting a pragmatic approach, where we focused on using process, performance and outcome-based goal setting using the (Specific, measurable, achievable, relevant, timely, evaluate, re-adjust) SMARTER structure (Healy et al., 2018; Locke and Latham, 2006)
The skill model (Dreyfus et al., 1986)	Shared Mental Models (Espevik et al., 2006)	Imagery: understanding the basics of imagery through the (physical, environment, task, timing, learning, emotion, perspective) PETTLEP model (Holmes and Collins, 2001; Lu et al., 2020)
Multi-action plan (Bertollo et al., 2016)	Adaptive skill (Ward et al., 2018)	Breath-work: learning the basic principles of applied diaphragmatic breathing (Ley, 1994)
Cognition and emotions (Lazarus and Folkman, 1984)	Community of practice (Li et al., 2009)	Positive self-talk and affirmations: learning the basics of how thoughts can influence behavior, learning to become conscious in how the internal dialogue can increase or decrease performance (Hardy, 2006)
Experiential learning (Kolb, 2015); Naturalistic decision making/different concepts (Klein, 2015)	Check-in/out (Smith et al., 2015); Feedback and feedforward (Hattie and Timperley, 2007)	Relaxation protocols: a basic understanding of relaxation protocols employed when using mindfulness (Hoyt, 2006; Meland et al., 2015)

The table describes the three-pronged approach, building on previous research (Ytterbøl et al., 2023) and adding in the theoretical components for performance development individually and for the team as a bespoke and comprehensive performance psychology educational training package.

The complete list of the content is presented in Table 1.

As Table 1 demonstrates, two main foci were selected: the team and the individual aspects of performance in their natural environment. The presenter was always provided a slot to guide the evaluation of the performance psychology content or activity. As with top athletes, the operators are experts in their respective fields. Accordingly, our approach to training was often to assist the operators by seeking ways to enable them to capitalize on their tacit knowledge (knowing more than they can tell) and to recognize the significance of this information by transforming it into explicit knowledge (Toom, 2012). To do this, we employed reflection on action, peer teaching, feedback, and peer-to-peer feedback.

Furthermore, along with promoting the philosophy of adaptive skills (Ward et al., 2018) and by exemplifying aspects from the psycho education curriculum [e.g., the Multi-Action Plan (MAP)]; (Bertollo et al., 2013, 2016), the presenter worked alongside the operators as he explored their chain of behaviors and cognitions that underpinned their process of performing optimally. Therefore, in line with Professional Judgement and Decision-Making Theory (PJDM) (Collins and Collins, 2017), the presenter's adaptability as a performance coach proved essential. Applying different theories and alternate modes of educational delivery—all with the aim of finding ways to make PP content valuable and meaningful to each operator were utilized. To present an example of how the training was conducted we have provided a sample of a training day.

### Sample of a training day

Usually, the day started with an informal reflection on the last 24 h and focused on the upcoming day. Training followed a semi-structured routine, which is exemplified in Table 2.

**Table 2:** Sample of a training day.

**Timings/task**	**Troop focus areas**	**Performance coach focus areas**
0500-0730 Physical training/breakfast	Maintaining physical standards	Being a part of the group
0730-0830 Team room	Plan for the day with specific focus areas. Admin	Sharing some aspects of performance psychology that could create a basis for learning (short lecture)
0900-1030 Close quarter battle (CQB)	Team and individual drills	Observe the training and participate in the feedback sessions, assisting them in exploring learning points both in teams and individual
**1100-1200 Lunch**		
1230-1430 CQB	Team and individual drills	Observe the training and participate in the feedback sessions, assisting them in exploring learning points both in teams and individually
**1530-1630 Dinner**		
1630-1730 Team room	Admin. Scenario brief	Repeat the most essential points from the day from a performance psychology perspective
1800-2100 Scenario training CQB	Putting their skills in context, specific scenarios	Observe the scenario training and participate in feedback sessions

As an example of lecture content, we introduced the concept of Shared Mental Models (Espevik et al., 2006), the potential of this construct for attenuating and syncing team members' shared awareness in training (i.e., Closed Quarter Battle), and how this could influence communication under pressure. Furthermore, we explored the impact this has on decision-making; we featured Recognition Primed Decision Making (RPD) (Klein, 1993), which explores and outlines a human-centric approach to decision making.

Each lecture was interactive. Opportunities were provided for operators to ask questions related to the performance psychology content and how the subject matter could be applied in a combat setting. Moving into the more scenario-focused/mission-specific training, the presenter's task was to explain the theory behind the subject, cite and consider practical applications, and to provide participants with signposts to where they could read more about the different subjects in the booklet provided. Following this, we worked through a practical example, inviting participants to test it out, quizzing them about where it would be applicable, and inviting them to reflect on this experience during their training.

### Data collection

Due to the longitudinal nature of the study (Yin, 2018), it became clear that more than one interview would be required. Early in the research process, and in part due to obtaining ethical clearance whilst also developing the semi-structured interview schedule, the scope of the research project became clearer. As the timeline shows in Figure 1, three interviews were conducted throughout the intervention with all seven participants, conducted in an informal, though private setting. All the semi-structured interviews were in-depth with a duration of 30–60 min. This allowed the interviews to be structured in a way that enabled respondents to be guided—whilst also affording ample opportunities to freely share their emergent thoughts and observations and to create personal meanings in response to each question (Levitt et al., 2017; Braun and Clarke, 2006). In addition, a follow-up interview with a member reflection process was conducted (Levitt et al., 2017).

All interviews followed the same procedure. Open-ended questions were posed, allowing the respondents to share form and share their responses and concurrently, the first author was afforded the opportunity to follow up on interesting data with probes and stimuli that were aligned with the overall purpose of the exploratory longitudinal case study. The first interview, prior to the performance psychology training commencing, focused on understanding important events in each participants' career. A timeline approach was used to try and uncover and understand transformative moments, starting with SOF selection and subsequently other elements in the training pipeline. The second part of the interview focused on their own thoughts around mental preparation for deployments.

Exemplar questions included:

- “Can you identify some important moments, like when you experienced in/out of control and significant events in your career and indicate when they happened?”
- “Could you describe your experiences with performance under pressure/handling of stress and how you prepare yourself for such situations?”

The second interview commenced after the work up period was finished, prior to deployment, and focused on understanding the operators' reflections after the work up, once they were familiar with the embedded performance psychology package.

Exemplar questions included:

- “To what extent did the training change or develop your mindset related to performing under pressure? Could you elaborate?”
- “What was the one lecture/training that you learned the most from? What did you learn specifically?”
- “In hindsight, what has been your most valuable experience in this training?”

The third interview was conducted after they had returned from the deployment and was subsequent to the operators' obligatory period of leave. The idea was that their experiences had matured, to a degree, and to then elicit further insights regarding their reflections around performance psychology for SOF.

Exemplar questions included:

- “To what extent did performance psychology training received, prior to the mission, help you perform on the mission? Could you elaborate?”
- “How do you perceive the culture in the unit when it comes to expressing vulnerability?”

Lastly, we conducted a member reflection and follow-up with each participant on a draft of the research paper.

### Data analysis

A Reflective Thematic Analysis (RTA), following the six steps (Braun and Clarke, 2019) was conducted to analyze the qualitative data, adhering to the Big-Q guidelines suggested by Braun and Clarke (2022) and from the overall 'big tent' criteria guidelines written by Tracy (2010). RTA is not a quantitative approach, and researcher subjectivity is a part of the research, which is “conceptualized as an art not a science” (Braun and Clarke, 2022, p. 9). This was especially important since the entire project focused on understanding the operators' experiences in relation to the intervention phase of this study (Braun and Clarke, 2019, p. 9), from a pragmatic standpoint.

After transcribing the interviews in Norwegian, they were subsequently translated to English. As a result, the familiarization process was immersive, with a special emphasis of maintaining the original meaning. The second step included inductive labeling (coding) capturing mostly semantic, but with latent notes from the transcription process on data relevant to research questions. This was a process repeated several times until all labels were printed out and compared, and subsequently reduced to generate clusters of shared meaning, which were then coalesced into themes and sub themes. This process was conducted to avoid what Braun and Clarke (2024) describe as topic summaries instead of generating themes. Once we began to write the paper, the first author spent further time understanding and implementing the ten guidelines as recommended by Braun and Clarke (2022) and decided to conduct tests where higher-order themes were traced back to their origin. This process resulted in changing some of the themes when the “reflexive interrogation” phase ceased (Braun and Clarke, 2022, p. 9). The initial analysis was conducted over 3 months, from September ‘21 to December ‘21. In addition, the first author's reflective diary (Braun and Clarke, 2019) which was established from the start of the data collection process, was maintained and added to throughout the data analysis phases; it became a welcome, trusted tool that enabled the first author to revisit specific journal entries, the meanings that were derived, and the consequent implications therein.

Moreover, its regular use resulted in building upon reflections during the data analysis stage. For example, the ability to go back and examine the notes for a particular session or event enabled reflexivity. The research team functioned as critical friends throughout this process (Smith and McGannon, 2018).

### Integrity, trustworthiness, and rigor

The first author has over 20 years of operational military experience. However, it was made explicit that, regardless of his professional background, the research aimed to understand what kind of PP package could assist the *operators'* performance in training and on missions. This was enacted by presenting different performance tools and letting the operators evaluate the concepts in their responses gleaned from the in-depth semi-structured interviews. Therefore, the first author played an active part in the production of knowledge. To harness this rich learning experience, two forms of reflexivity were utilized: introspective reflexivity, understanding the position in relation the context of the study, and intersubjective reflexivity—the relationships with the participants (Finlay, 2002). In interviewing operators that the first author had trained and lived with for an extended period, there was several important aspects to consider.

Whilst focusing on maintaining the perspective on the operators' unique experiences, the first author was acutely aware of conducting a PP program in which he, as the researcher, was heavily invested. This necessitated taking extra care not to impose his own thoughts and ideas onto the operators (McGrath et al., 2019). Another important consideration regards “going native” (Bryman, 2016, p. 394). Although a native in the big picture within the NAF, the first author had not served with any of the participants, nor in the same capacity. Therefore, his native-ness enabled access to this hard-to-reach special population, but did not confound the consideration of the data. Consequently, obtaining access to this performance domain, contributing to the knowledge held within it, and having access to the data derived from it, affords the first author a privileged position that offsets any perceived benefits of assuming an objective position—in relation to the phenomena in question (McGannon et al., 2021). Finally, since the project was conducted in Norwegian, the member reflections were conducted in English, thereby authenticating the original meaning from the participants themselves as opposed to a back translation process (Behr, 2017).

## Results

**Figure 2:**
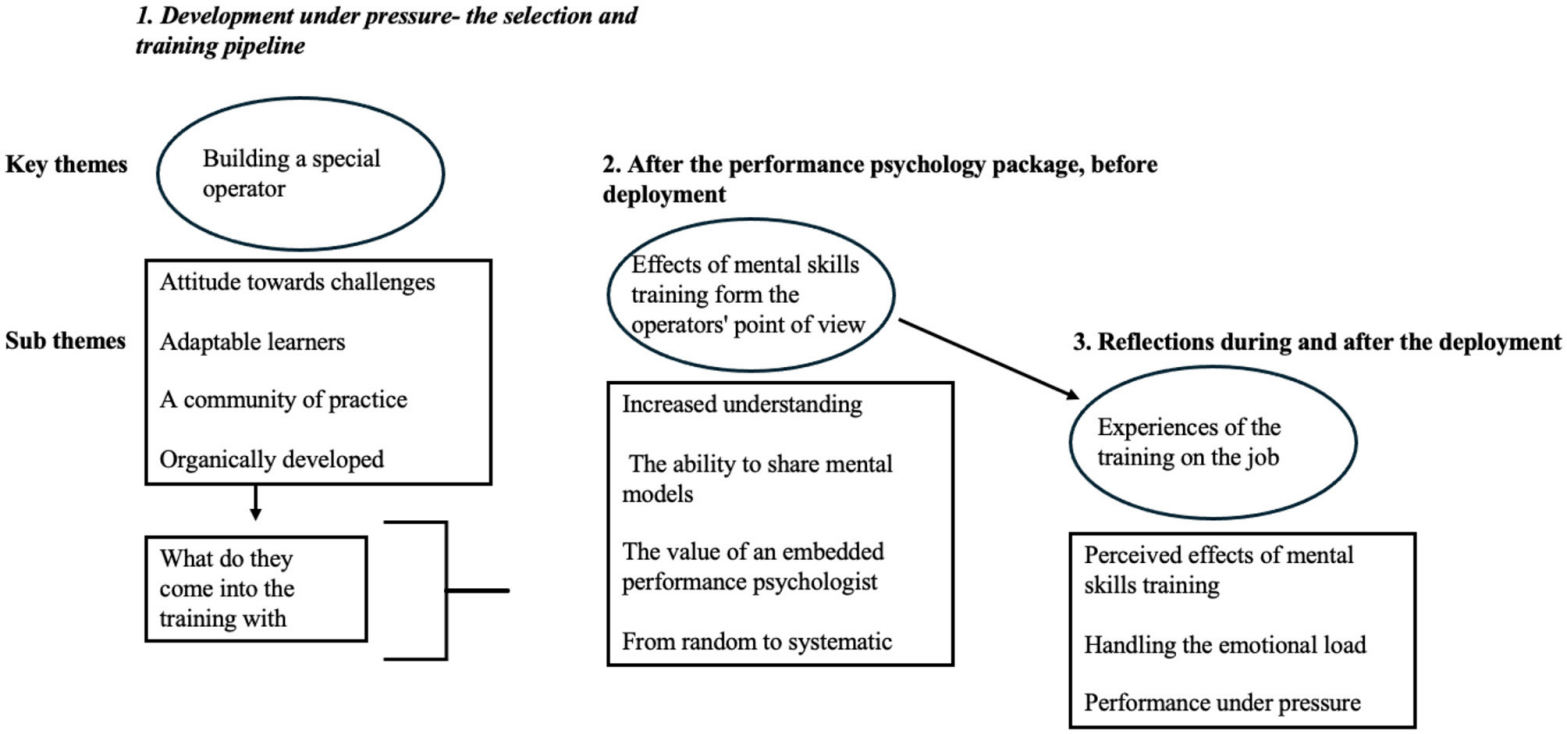
Overview of the themes and sub-themes in the thematic analysis.

In order to present the results coherently, Figure 2 presents the three main themes and sub themes. The first presents a thematic analysis before the PP. The second and third theme presents the analysis after the training before the deployment and after the deployment.

The first key theme is the result of the Reflexive Thematic Analysis conducted before the training package commenced (Figure 1). This encompasses how their development took place under pressure. All participants commented on how they experienced their mindset prior to the selection:

1. **Development under pressure- the selection and training pipeline (****Table 3****)**.

**Table 3:** Key theme: building a special operator (*n* = 7).

**Sub-theme**	**Raw data exemplar**
Attitude toward challenges (*n =* 7)	“There was no way I was going to quit. So, I turned it around and thought like this, it is impossible not to make it, right! …I am intelligent, my fitness is good, I am not going to quit. …People psyche themselves out when they are alone in the tent. Instead of, like, just put down the work and don't quit.”
Adaptable learners (*n =* 7)	“During the selection, my thought process was that no matter how terrible it becomes, it will only last a certain time. …and this is a good thing, because I learned that no matter how many bad periods, it fluctuates a lot. And this is a good thing because I have learned that the hard periods, they pass.”
A community of practice (*n =* 7)	“The most important (factor) for us is training together so that we are a team, everybody knows each other's strong and weak sides. That we know each other in a way so we can function well together. That team stuff is important for us to work together because that is what we are going to do.”
Organically developed (*n =* 7)	“It is through training that you build confidence and all those things. …I have not actively conducted any mental training, and I don't feel we have had a focus on it, but I do not feel that it has been missing either. …I have never been deployed and didn't feel prepared.”

Results suggest that this unit are very good at what they do, at least according to their own perceptions. Importantly, this is supported by their status within the Norwegian military milieu. The key theme, “building operators”, encompasses some of the skills developed during this research study; specifically, selecting the right people and training them to unit standards:

Parts of the selection was a rollercoaster mentally. …The bergens were extremely heavy and the marsh was wet. I thought to myself, it is now or never, I don't care anymore. …Then on another evolution, it was a blizzard in the middle of the summer, and a gust of wind came upon me, there I was with snow in my face and looked out over the terrain. I felt like a lion on top of that mountain. Goosebumps. I knew I was going to make it. Like a surge through my body. I knew I was going to make it. I believed in myself, I just grinded through (E).

(E) describes becoming indifferent and perhaps almost giving up. Still, he pushes through.

Interestingly, as displayed in Table 3, we see various organically developed mental skills that surface. For example, in the last sub-theme, participants do not feel that something is missing, but they always focus on getting better. Continuing with the words of (E), “We will never be perfect, but we must always chase becoming perfect”. Furthermore, (B) describes, “Lot of what we do in our work is like a mental preparation. We are deliberately put in situations or scenarios we might encounter”.

In sum, through their selection and training, the operators reach a very high level of performance. Clearly important for the psychosocial aspects of their performance (e.g., pressure to perform and social support through shared experience) we would suggest that this also made the operators more open to the intervention. In simple terms, already confident in their personal standards, they were more open to anything which could make them even better. This is an important characteristic which needs careful presentation to such groups so that it can be fully exploited. The first author's background and credibility would appear to be crucial in this regard.

**2. After the performance psychology package, before deployment**.

Presented here (Table 4) is the immediate impact of the performance psychology curriculum applied to the operators during their workup period (Figure 1).

**Table 4:** Key theme: performance psychology from the operator's point of view (*n* = 7).

**Sub-theme**	**Raw data exemplar**
Increased understanding (*n =* 7)	“I feel like I have done some of this before, subconsciously, and through this training I feel like I have gotten a confirmation that what I do is correct. I don't feel like I learned any like revolutionary stuff, but it is good to put it into words and it makes it easier to explain it to others, which has been difficult earlier”.
The ability to share mental models (*n =* 7)	“Now it has become something that is the back of the head, either because you come to the whiteboard a couple of times during the day or remind us of it, okay, then it is with us a couple of sessions, then you forget some, fall back to old habits, then you learn something new, and then I take some more time and put some extra effort in my preparations, before I go through the task and make sure everyone shared the same mental models”.
The value of an embedded performance psychologist (*n =* 7)	“For me personally, it has been very good. I believe the most important thing is that you are here, and we trust you, which makes it flow in day-to-day training. To be able to do it in a way that doesn't remove focus from our primary tasks but integrates the training into it. The most important thing is that we can focus on it, we haven't had anything with that kind of focus before”.
From random to systematic (*n =* 7)	“Achieving a clearer mental picture of what I want to achieve, sensing my breath, my focus on that part has increased, and the most important part, I am repeating the sequence every time. As opposed to previous episodes, where it was random”.

Analysis indicates how the operators experienced the PP training as a positive learning experience, exemplified through the sub-themes. Individual perceptions of the most important part of MST differ. To exemplify, operator (A) stated:

I expected that I would get some confirmation on the stuff I already do. That is a good feeling. Just as an example, with working memory overload. I have never had a word for it. …like a confirmation that I do the right things (A).

(A) brings forth a specific example from the PP package: namely, working memory overload. The training gives him a confirmation of the fact that he is doing the right things (making tacit knowledge explicit). Turning to operator (G):

I feel like I have developed myself mentally and have grown in several years [in experience] in a very short time. It comes back in everything I do. I do not need much time to focus; my decision-making is sped up because I have confidence in my abilities. I experience more control in chaotic circumstances than I experienced earlier. I know what type of reactions are natural for me and that what I interpreted as fear is just awareness of my surroundings. This has led to improved confidence in demanding and dangerous situations. I feel good, I feel good now. In everything I do. Confident and in control (G).

(G) describes how he has been able to integrate the training into his practice holistically.
**3. Reflections during and after the deployment**.

This section covers reflections and perceptions based on operators' data collected post-deployment (Figure 1; Table 5).

**Table 5:** Key theme: experiences of the training on the job (*n* = 7).

**Sub-theme**	**Raw data exemplar**
Perceived effects of mental skills training (*n =* 7)	“What can I say? It contributed, but it is an extreme performance culture already, how much more it has evolved, I cannot give you any exact numbers on, but that the focus has increased on these things is without a doubt. A lot. So, I believe we were good at maintaining our focus on the important tasks when the situation was what it was, and this stuff [MST]has influenced our ability to do that, I certainly believe so”.
Handling the emotional load (*n =* 7)	“Hmmm. It seems like the newer guys have started to open earlier than before. With the guys who I have served with for 10 years or so, we know each other well. So, it is what we wanted, to understand this earlier, maybe it happens earlier now …it is no “pride” now, if you know what I mean. It is the way it should be”.
Performance under pressure (*n =* 7)	“I assisted in a medic case, and I was very conscious of the fact that I wanted to calm myself down and disregard everything else in the situation. I did not focus on my breathing initially, but on relaxing my muscle tension lower my heart rate as much as possible, very conscious on that part, not letting myself be influenced. It helped to focus on my breathing and being aware of my emotions”.

When it comes to the first sub-theme, the experienced effect of MST differs. Operator (B) reflects on the completion of their mission and attributes the outcome, in part, to maintaining their readiness status; [the training provided pre-deployment] “It is a major contribution to being professional. Increase in confidence, skills, both socially and what we do”. Operator (D) states; “Ehmmmm. We are a bit more conscious now. …worked well together in the troop. Much of the stuff we went through [the training provided pre-deployment], we increase our performance, for me, especially those SMM [shared mental models], things become more natural” [in their communication] (D).

When it comes to handling the emotional load, Operator (G); “We share more than we used to”, and he believes,—that when *the* situation occurs—the enhanced interrelatedness is viewed as an essential factor; it is the human aspect that governs the efficacy of the response—and in turn the outcome.

Another vital aspect raised was regarding mental health (F):

I experience talking about mental health as a taboo. I feel that we have removed a barrier by talking about this. I believe that is very important in a group going in(to) combat together. It has become more natural to talk about these things that affect us daily. Big, strong guys are not supposed to have feelings. If something bothers you, it is seen as a weakness, and everybody wants to uphold their image of being strong with a degree of confidence. We have high confidence, but talking about emotions to perform better is very important (F).

When it comes to the last sub-theme, performance under pressure, there are indications of an increased and more considered pattern of cognition in how the operators approached stressful situations by focusing on the task at hand and greater awareness of their psychophysiological state with a view to maintaining and optimizing performance.

**4. Follow-up and member reflections**.

To understand more about how the operators experience training, a follow-up was conducted approximately 18 months after the case study was finished. To conduct the follow-up such a long time after the interventions holds the potential to add value to the investigation, issues of recollection notwithstanding. A complete version of the paper was sent via encrypted email to each of the participants, with their individual codenames, the questions attached, and 3 weeks to read through the present study before the first author followed up with a voice call. All the respondents (*n* = 7) opted to answer.

1. Any comments on the paper?

In sum, the operators thought it was interesting to read, and it provided an accurate description of the training package.

(A): “Very professional and easy to read. It is recognizable, the translations are spot on, and a solid description of the program”.
(G): It is to the point and easy to read. It was fascinating reading about the other participants and how they described their thoughts and strategies. I also recognize myself in their descriptions. I am curious to see the conceptual development of this further and how to integrate performance psychology into daily training.
(C): Your conclusions and opinions in the paper mirror my own experiences of the program. I remember it as exclusively positive; it was not creating more noise or extra work in a busy work up period. Another factor is that we as a group had the possibility to train for a mutual goal—the mission—which I think was an important factor regardless of the performance psychology interventions. At the same time, I believe that what you did was not an insignificant contribution. We discussed a lot of the stuff we went through, also when you were not there, and we would not have done this if it didn't produce an added value.

2. Today, with regard to the PP package what do you regard as the most valuable element for you?

Operators experienced and emphasized the value of learning PP in a systematic manner and felt this was important. They also commented on how they picked up on various elements individually and developed their own interpretations in the context of their professional experiences.

(B): Looking back at what we did today, I still believe this was an excellent awareness and training for most of the troop. As you describe, for some, this was a tacit-explicit thing, that our methods and way of thinking were good. For others, it represented learning methods that they did not know before and were helpful for them.
3. Any additional comments or things you want to say?

In this section, a few operators had some thoughts:

(F): “I particularly liked what you wrote about the focus you had in not influencing us in the interview, which could have been easy to do since you were so integrated into the group”.
(E): “Only last night I thought about the priming we did before Close Quarter Battle (CQB) training.”
(G): “Yesterday, I found the booklet and read about shared mental models again.”

To summarize their answers, it seems clear that the performance psychology package was received as a positive contribution to their training and that they continue to utilize parts of the constructs presented.

## Discussion

The purpose of the investigation was to:

1. Offer an evaluation of integrating a bespoke performance psychology package on SOF personnel against the challenges of immediate mission preparation and longer-term impact.
2. Evaluate the perceived effect of mental skills training on already well-established elite performers, both immediate and post-deployment—and in the longer term.

Based on the results from the first key theme, *building a Special Operator*, essential aspect of this research was to understand more about what the operators arrived with, the aspect of “not giving up” could be regarded as a natural cornerstone for these operators. Without this capacity, they would not continue after the initial selection, nor through the training pipeline. Perhaps it can be regarded as both a foundational skill and to an extent a personality trait. Its implications, based on Operator (E) example from selection implies this is what sets SOF apart. Consequently, it is viewed as a resource that the operator draws upon when experiencing grueling mental and physical challenges.

Furthermore, the evidence we have collated and analyzed suggests that the development of specific mental skills occurred as a concomitant factor associated with operators' selection and training. Given what the operators are required to undertake during selection and other phases of their careers, it is not surprising they have developed psycho-behavioral characteristics of excellence (MacNamara et al., 2010) and the resultant capacity for elite performance as a learned and adaptive coping strategy. This indicates that operational SOF are adaptable experts (Cruickshank et al., 2020; Mees et al., 2020; Ward et al., 2018) who are shaped and developed by their communities of practice (COP) (Li et al., 2009).

Our research suggests that how the operators approach challenges and cope with different types of stressors is vital to their success in passing the selection—and—once selected, they are required to continually perform to a high standard in the subsequent program of training and deployment. Therefore, their journey can be explained through the development of personal self-efficacy because “what you believe you can do with what you have under a variety of circumstances” (Bandura, 1997, p. 37) shapes our perceptions of the task. Clearly, it is not so much about who you perceive yourself to be at the start of a SOF career—it is the acceptance of what is occurring and what is likely to occur in training and on operations, combined with a mindset of perseverance, and what could be described as the development of mental skills—through adaptation, mimicry, and innovation.

Concerning the second key theme, *the effects of mental skills training from the operator's point of view*: Against our primary objective, the integration of bespoke PP in an embedded form made a valuable contribution to the operators' training. Examining the follow-up, the narrative evidence indicates that the perceived value was immediate and consistent; furthermore, it evolved organically over 18 months. The results indicate that the operators experienced an increase in their understanding of mental skills and how to use them. There are two components at play here: expressing what you know and how you have realized your talent is challenging to articulate. We suggest that an integral challenge of working with elite performers/operators for practitioners and researchers is finding the means to enable them to elicit the tacit knowledge *(knowing more than you can tell)* they have acquired (Collins, 2010), which in-turn increases the capacity to problem solve and embed and enrich knowledge in communities of practice; and secondly, this effect is amplified by the MST training conducted as a part of their training. The potential challenge of transfer from knowledge to practice has been highlighted in previous research (e.g., Koerner and Staller, 2021) but, in the present case, we would suggest that our data from the fourth interview (at follow up) supports that this has taken place. The operators do seem to have reflected on, experimented, then selectively applied aspects of the PP education program to their subsequent practice. Indeed, we see the assurance of this transfer (a major goal of the intervention) as the primary reason for incorporating a longer term follow up.

Concerning the whole PP package and how it was taught, the operators highlight different, though complementary, aspects that made sense for them (Collins et al., 2022)—and that an embedded practitioner approach facilitates their learning. The experiential learning theory (Kolb, 2015) provides a methodological approach that fits naturally into the existing training environment and performance culture of this under-researched population. When this method is combined with a systematic coaching concept (delivery system) that caters for individuality, but at the same time follows a structure, this can directly enhance the training output (Collins et al., 2022). Furthermore, an embedded approach allows for greater precision in applying and adapting content, both individually and for the team. This is perhaps identifiable by operators' shared experience of being able to explicitly communicate with the practitioner/researcher, thus increasing their ability to share and make sense of their mental models (Espevik et al., 2006). The operators work in a practical environment on a wide range of tasks; therefore, we contend that future best practice for this sub-group of operators would be to have a performance coach present “in the wild” for extended periods at a time, to facilitate the operators' psychological performance as they embark on their respective journeys—bridging theory/practice.

When it comes to the different MSTs taught, the importance of delivering a “deck of cards” (cit. Collins and MacNamara, 2017) is further enhanced; according to this approach, each operator has different needs, skills, preferences and attitudes: correspondingly, the *in-situ* psychologist needs to adjudge which skills, in which order, and to whom the “cards” are dealt. The benefits of this approach are that the operators learn mental skills systematically, as opposed to it being a series of scattered, fragmented factors that are poorly connected which could result in an ill-developed mental schema. Several factors share similarities with research we have conducted on a comparable group, utilizing a similar MST setup (Ytterbøl et al., 2023). On the one hand this reinforces the results in this paper. On the other hand, it could indicate a bias toward our own research. Lastly, their natural way of training, linked to various constraints occurring ecologically, perhaps enhances their ability to integrate PP in a practical way.

In the last key theme, *Experiences of the training on the job*; through our analysis of the operators, the unit's operators display reflective and metacognitive abilities; in other words, they think clearly about their thinking (Veenman et al., 2006) regarding their training tasks and missions; moreover, they articulate themselves clearly on how the effects of the MST curriculum equipped them to deploy.

As pracademics working in this context, we found it useful to introduce a theoretical approach to the process of decision-making under stress, and more specifically, Naturalistic Decision Making (Klein, 2015). The participants' ability to perform under pressure is already at a high level having been developed through selection and training. Firstly, having a solid empirical theory as a foundation, such as Recognition Primed Decision Making (RPD) (Klein, 1993), which explores and outlines the implications of making decisions under time pressure and uncertainty (Klein and Wright, 2016), further allows operators to interpret and build upon their own experiences. Importantly, RPD posits that intuitive decisions are based on previous experience and training. In turn, this enables experiential learning and training to engender the requisite levels of cognitive capacity necessary to operate and survive difficult situations. Furthermore, the individual operator can utilize self-regulation techniques such as controlled breathing (Jerath et al., 2015) and self-talk (Hardy, 2006), using psychophysiological and cognitive techniques to enable effective decision-making and at the same time ensuring that their brain/body system is prepped to execute the skill optimally.

Secondly, results suggest that increased awareness and understanding of the individual operator and their team's current skill level could assist in the development and optimization of the unit's current training regime—and their preparation and planning. Clearly, there is an apparent link to Dreyfus and Dreyfus' (2005) five stages of expertise development; precisely and concerning this model and exploiting the link between theory and practice—operators can build upon and extend the culture and established COPs that we contend are inherent in SOF units (Li et al., 2009).

The fact that the operators reported that they performed better, both as a team and individually, after a period of focused reflection and PP training should not come as a surprise. The more contentious component is the *system* of delivery—specifically, the status and experiences of the practitioner who delivered the training. A further stimulus to performance could have been derived from the first author's operational experience. SOF, for obvious reasons, is a challenging environment for practitioners to operate in—but as a detached observer, but with experience of operating in a military environment, access was afforded, and conversation perhaps flowed more readily—than it might with a bona fide “outsider”.

Whether this is typical of all SOF groups or just this one is moot; there is an apparent acceptance of comrades sharing their perspectives and emotions concerning training, operational, and personal matters. These social and cognitive factors can contribute to enhancing individual and group skill acquisition chain (Dreyfus et al., 1986; Dreyfus and Dreyfus, 2005; Hoffman, 2013) by increasing awareness of their respective roles in employing complex mental models (Espevik et al., 2006). Furthermore, the operators outline examples of integrating different elements from the performance psychology package into their toolbox and underline the importance of their individuality in the process. Finally, the follow-up reinforces the initial results, especially as operators are already committed to actively work on their continued learning and understanding of the performance psychology package.

All studies hold limitations; there was a low number of participants, yet the population pool, given the high level of expertise was necessarily small. We have not used a control group—given that the investigation was undertaken in a highly dynamic environment making it impractical to do so. It was based on self-report and observational data, and the expectancy effect of the interventions must be allowed for, especially since the first author spent so much time integrated with the team. However, as operator (F) highlights in the follow-up, the process was objective and that goes some way to addressing this shortcoming. Despite no suggestions for improvements being voiced by the operators—it does not mean there is no room for improvement in the delivery and design of the training package and the study as a whole—especially since it is a pilot study. Furthermore, no quantitative data were obtained.

Lastly, the philosophy, pedagogy and delivery of the performance psychology curriculum are not standard in the Norwegian Armed Forces, meaning the novelty of the interventions itself could have influenced operators' motivation and interest to perform. In sum, further research is needed, and it could use this pilot study as a foundation, to go more in depth on different aspects and utilize a mixed methods approach with triangulation.

## Conclusion

Based on our research, we argue that proper case conceptualization starts from considering the operators' selection and training pipeline—and the experiences and skills that it engenders. Developing a bespoke performance psychology package in line with the unit's culture and ethos, whilst also making allowances for and recognizing individual differences is a crucial step (Martindale and Collins, 2007). With regards to the *special*ness of SOF, and the fact that optimum performance is sought through bespoke solutions developed organically further validates the operator-centric approach of conducting training and research with them and for them—not on them (Ytterbøl et al., 2022). Finally, it provides an example from this particular “laboratory” of top performers. It indicates PP can be a beneficial tool—for already high-performing teams and individuals.

## Data Availability

The original contributions presented in the study are included in the article/supplementary material, further inquiries can be directed to the corresponding author.
